# Controlled Measurement Setup for Ultra-Wideband Dielectric Modeling of Muscle Tissue in 20–45 °C Temperature Range

**DOI:** 10.3390/s21227644

**Published:** 2021-11-17

**Authors:** Gertjan Maenhout, Tomislav Markovic, Bart Nauwelaers

**Affiliations:** 1 Division Telemic, Department of Electrical Engineering (ESAT), KU Leuven, Kasteelpark Arenberg 10, Box 2444, 3001 Leuven, Belgium or tomislav.markovic@imec.be (T.M.); bart.nauwelaers@esat.kuleuven.be (B.N.); 2 Imec, Kapeldreef 75, 3001 Heverlee, Belgium

**Keywords:** biological tissues, dielectric measurement, dielectric model, measurement metadata, muscle tissue, open-ended coaxial probe, temperature, ultra-wideband

## Abstract

In order to design electromagnetic applicators for diagnostic and therapeutic applications, an adequate dielectric tissue model is required. In addition, tissue temperature will heavily influence the dielectric properties and the dielectric model should, thus, be extended to incorporate this temperature dependence. Thus, this work has a dual purpose. Given the influence of temperature, dehydration, and probe-to-tissue contact pressure on dielectric measurements, this work will initially present the first setup to actively control and monitor the temperature of the sample, the dehydration rate of the investigated sample, and the applied probe-to-tissue contact pressure. Secondly, this work measured the dielectric properties of porcine muscle in the 0.5–40 GHz frequency range for temperatures from 20 °C to 45 °C. Following measurements, a single-pole Cole–Cole model is presented, in which the five Cole–Cole parameters ($\epsilon_{\infty }$, $\sigma_{s}$, Δϵ, τ, and α) are given by a first order polynomial as function of tissue temperature. The dielectric model closely agrees with the limited dielectric models known in literature for muscle tissue at 37 °C, which makes it suited for the design of in vivo applicators. Furthermore, the dielectric data at 41–45 °C is of great importance for the design of hyperthermia applicators.

## 1. Introduction

Since the discoveries from H.P. Schwan last century [1], the dielectric profile of biological tissue has been widely studied and has led to the observation of four dispersion regions. The large increment at low frequencies has been ascribed to the α dispersion mostly due to counterion polarization [2,3,4]. Next, the cellular and intracellular membranes in tissue limit the flow of ions in tissue, and, as a result, charges are accumulated at these membranes. This causes interfacial polarization that is visible in the β dispersion [5,6]. In the ultra-high frequency (UHF) and super-high frequency (SHF) range, the dielectric profile is shaped by the γ dispersion as a result of the presence of the water molecules in tissue [7]. This γ dispersion is strongly correlated with the water content of tissue [8,9]. Lastly, a fourth dispersion has been noticed. This δ dispersion is located in between the β and γ dispersion. Its origin is not yet fully understood, but it should be related to the interaction of water molecules with other molecules. This interaction imposes a restriction on the mobility of water molecules and results in a decreased relaxation frequency [10,11]. The use of these dielectric properties led to the development of multiple electromagnetic (EM) applicators in multiple frequency bands, e.g., electrical impedance tomography [12], radiofrequency (RF) tissue ablation [13], and microwave tissue detection [14], hyperthermia [15], and ablation [16].

**Figure 1:**
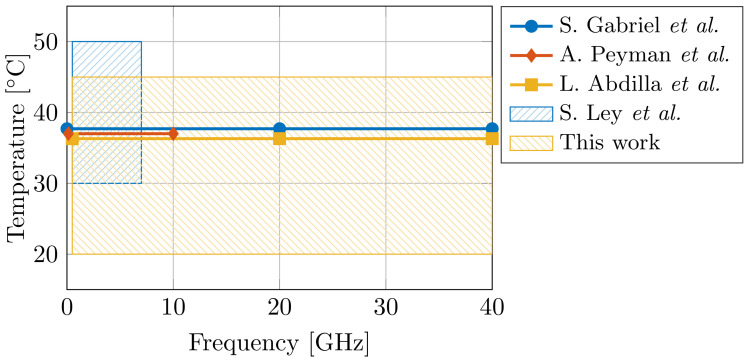
Comparison of the operating temperature and frequency range of dielectric models for muscle tissue [19,22,23,24] from state-of-the-art literature.

The design and the effectiveness of said EM applicators is directly correlated with the knowledge of the dielectric properties of the tissue. Therefore, dielectric tissue databases are proposed to increase the ease of access for EM designers. In 1996, S. Gabriel et al. presented dielectric data for 17 tissues [17,18,19], and, in 2011, an online database containing 108 tissues was presented by the IT’IS Foundation [20]. However, ultra-wideband dielectric data in literature is still scarce for a broad temperature range. Given the aforementioned importance of water on the dielectric properties of tissue at the γ dispersion, and given the temperature-dependent dielectric response of water [7], the influence of temperature on dielectric tissue measurements is major. A difference of a single °C can result in a 2% change in dielectric properties [21]. Therefore, more ultra-wideband temperature-dependent dielectric data is required. In the case of muscle data, only three dielectric models are known at 37 °C (S. Gabriel et al. [19] for 10 Hz to 100 GHz, A. Peyman et al. [22] for 0.13–10 GHz, and L. Abdilla et al. [23] for 0.5–40 GHz), and a single model covers the 30–50 °C temperature range (S. Ley et al. [24] for 0.5–7 GHz). Figure 1 demonstrates the limited operating temperature and frequency range of aforementioned models. Thus, the first objective of this work is to present an ultra-wideband dielectric model of muscle tissue, valid in a broad temperature range.

Furthermore, the impact of external confounders on dielectric measurements of biological tissue is not limited to only tissue temperature. Throughout the past decades, multiple confounders influencing dielectric measurements have been identified. These can be originated back to the tissue sample itself, e.g., the age of the animal of which the tissue was harvested [22], the time between the death of the animal and the measurement [25], and the possible heterogeneity of the sample [26,27]. However, the used measurement setup and techniques can have an influence, as well. Firstly, the combination of an increased temperature compared to room temperature and a time period for sample preparation and measurements makes the tissue sample prone to dehydration [28,29]. This can result in a difference in permittivity of up to 9% for measurements at body temperature during 35 min [30]. Secondly, applying a variable probe-to-tissue contact pressure with the most-commonly used slim-form open-ended coaxial probe can result in a −0.3% change in permittivity per kPa [31]. In addition, the minimum dimensions of the sample under interest have to be respected [27,32]. Therefore, measured dielectric data should be obtained with a standardized measurement setup as proposed by the MINDER framework [33]. Thus, the second objective of this work is to present such a controlled measurement setup for dielectric measurements of biological tissue.

This work will start with the presentation of the used controlled measurement setup for dielectric measurements of biological tissue. Given the influence of temperature, dehydration, and probe-to-tissue contact pressure as discussed in our previous work [30,31], a controlled measurement setup for dielectric measurements of biological tissue is presented in Section 2. To the authors’ best knowledge, this setup will be the first one to actively control and monitor the dehydration rate of the investigated sample, the temperature of the sample, and the applied probe-to-tissue contact pressure. Furthermore, other relevant environmental conditions, such as room humidity and room temperature, will be observed and reported, as well. Next, dielectric measurements are performed on the muscle samples in the 0.5–40 GHz frequency range for temperatures from 20 °C to 45 °C, and Section 3 presents the results. This range renders the model useful for dielectric properties at room temperature, body temperature, and hyperthermia temperatures, and it includes three industrial, scientific, and medical (ISM) frequency bands. In Section 4, the measurements are fitted to a Cole–Cole model and compared to data available in literature. Lastly, the improvements of the controlled measurement setup and the relation between the obtained dielectric measurement results and literature data are compared and discussed in Section 5.

## 2. Materials and Methods

### 2.1. Porcine Muscle Samples and Sample Preparation Procedure

Measurements are performed on porcine muscle tissue. The samples were purchased at a local butcher where they arrived the same day from the slaughterhouse. Afterwards, they were stored in a fridge at 7 °C to prevent tissue degradation. Recent studies demonstrate the negligible influence of storage on dielectric measurements at frequencies above 1 kHz [24,34,35]. Experiments were performed at eight different temperatures from room temperature up to hyperthermia temperatures with special attention for body temperature (20, 25, 30, 35, 37, 40, 43, and 45 °C). Before every round of measurements at a single temperature, three samples of 2 cm × 2 cm × 2 cm were freshly cut off. The tissue samples were stored in sealed plastic bags and placed in a water bath (TSGP2S, Thermo Scientific, Waltham, MA, US) to heat them to the desired temperature. Once the sample temperature stabilized over two minutes, the sample was taken out of the water bath, dried with a paper towel to prevent excess moisture at the probe tip, and transported to the measurement setup. However, due to the transportation from the water bath to the measurement setup, the actual sample temperature deviated from the desired temperature. Therefore, the temperature at the moment of measurement was recorded and will be used in the remainder of this work.

### 2.2. Controlled Measurement Setup for Dielectric Measurements of Biological Tissue

**Figure 2:**
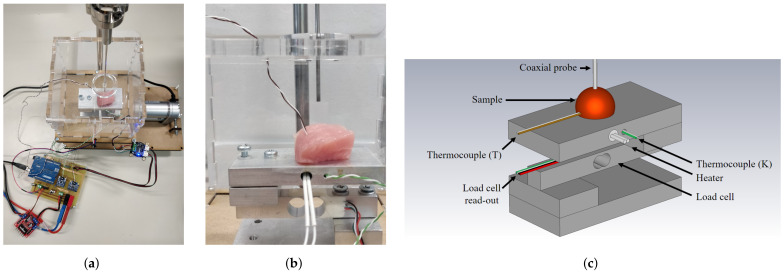
(**a**) Photograph of the controlled measurement setup. (**b**) Detailed photograph of the setup with a sample on the heated platform enclosed in a PMMA enclosure, ready to be probed for dielectric measurements. (**c**) Labeled, graphical representation of the measurement setup.

The measurement setup is built upon our previous work with a strong focus on actively controlling and monitoring the temperature of the sample, the dehydration rate of the investigated sample, and the applied probe-to-tissue contact pressure [30,31]. After the aforementioned heating in the water bath, the sample is placed on the heated platform, as shown in Figure 2.

This additional heating of the platform helps to maintain a stable temperature throughout the dielectric measurement. The sample and the heated platform are placed in a polymethylmethacrylate (PMMA) enclosure to reduce the air flow around the sample and, thus, reduce the corresponding dehydration [30]. As explained in Reference [31], the platform is placed upon a PI-controlled lifting platform to ensure a stable probe-to-tissue contact pressure as opposed to manually pressing the sample to the probe. Here, we opted for a contact pressure of 15 kPa as this gave us the lowest standard deviation in our previous work. Furthermore, a similar contact pressure for dielectric measurements was recently proposed by A. Martellosio et al. [36]. Additionally, a humidity and temperature sensor (HDC1080EVM, Texas Instruments, Dallas, TX, USA) measured the relative room humidity and room temperature. Lastly, each sample was weighed before and after the experiment to grasp the influence of dehydration throughout the time period for sample preparation and dielectric measurements at different temperatures.

The dielectric measurements were conducted with a slim-form open-ended coaxial probe (N1501A, Keysight, Santa Rosa, CA, USA) and a vector network analyzer (VNA) (PNA E8361A, Keysight, Santa Rosa, CA, USA), as explained before. The power level of the VNA was set to −5 dBm to ensure that the input power does not induce any thermal effect in the sample as demonstrated before by our group for different microwave for life science applications [37]. Therefore, the dielectric measurement itself will not influence the sample temperature, nor will it contribute to tissue degradation. Measurements were executed from 500 MHz to 40 GHz with a frequency step of 25 MHz, and an intermediate frequency bandwidth (IFBW) of 300 Hz was used. Before and after all measurements at a single temperature, a pre- and post-validation measurement was conducted on a 0.1 M NaCl solution at room temperature and the standard deviation of the measurement was calculated to obtain the random uncertainty, systematic uncertainty, and drift, as explained in Reference [30]. Despite the similarity between the saline solution validation liquid and the distilled water (DIW) standard, its use is nevertheless widely accepted as a reference due to its stability at various temperatures and its ease of storage compared to alcohols [23,38,39,40,41].

## 3. Measurements Results

### 3.1. Stability of Controlled Measurement Setup

**Figure 3:**
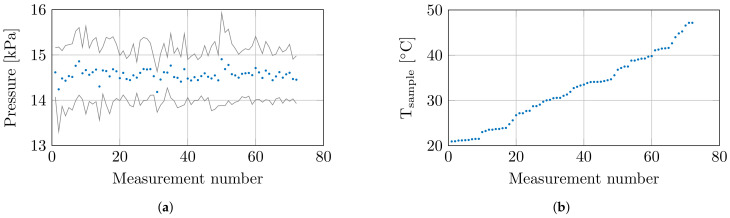
(**a**) The mean contact pressure at each measurement with ±1 standard deviation. (**b**) The recorded sample temperature at each measurement.

At the 8 set temperatures, 3 samples were measured at 3 locations, which resulted in a total of 72 measurements. Throughout these 72 measurements, the environmental conditions and confounders were closely monitored. The humidity and temperature sensor measured a relative room humidity of 71.4 ± 0.9% and a room temperature of 20.6 ± 0.4 °C throughout all measurements. The controlled measurement setup probed the tissue sample with a controlled probe-to-tissue contact pressure of 14.6 ± 0.61 kPa, and the average contact pressures with their corresponding standard deviation are shown in Figure 3a. Furthermore, the actual sample temperatures are shown in Figure 3b.

Lastly, the relative weight loss of each sample was calculated as a guideline for sample dehydration. Due to the sealed bags in the preheating phase, the limited time in air, and the limited exposure to air flow in the PMMA enclosure on the controlled measurement setup, an insignificant relative weight loss below 1% was observed for samples heated up to 40 °C. However, at higher temperatures, the relative weight loss increased up to a maximum of 6%.

### 3.2. Dielectric Measurement Results of Muscle Tissue

**Figure 4:**
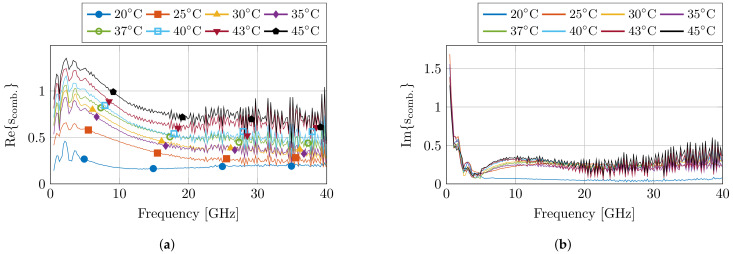
The calculated combined complex uncertainty $s_{\mathrm{comb}.}$ for each set of measurements at the corresponding temperature step. The real and imaginary part are displayed in (**a**,**b**), respectively.

Before and after the dielectric measurements at each set temperature, a pre- and post-validation measurement was conducted to calculate the standard deviation of the measurements. This resulted in the combined complex uncertainty $s_{\mathrm{comb}.}$ for each measurement run at a set temperature, which is presented in Figure 4. The uncertainty values are in line with previous experiments. Nevertheless, an increase in uncertainty is visible towards later measurements due to drift of the calibration, but the obtained results are still within reason.

**Figure 5:**
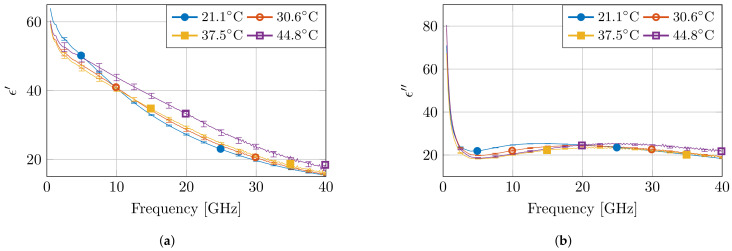
The measured dielectric properties at four temperatures with the corresponding standard complex uncertainty $\pm \;1\;s_{\mathrm{comb}.}$ for each measurement. The real and imaginary part are displayed in (**a**,**b**), respectively.

Figure 5 presents the measured dielectric properties of four measurements at different temperatures, together with the calculated standard complex uncertainty value $s_{\mathrm{comb}.}$. Due to the the non-overlapping error bars for multiple measurements, the observed differences in temperature at fixed frequencies are statistically significant. Moreover, as expected from a high-water content tissue, such as muscle, the dielectric profile follows the behavior of water as a function of temperature [7]. At low frequencies, the permittivity value is higher for low temperatures. Next, a crossover zone appears, after which the permittivity value is higher for high temperatures. Furthermore, the measurements at 45 °C stand out due to a larger change in permittivity compared to other temperature steps. Nevertheless, a similar behavior is observed, as well in other high-water content tissues, such as liver, for similar temperature changes in a reduced frequency range [21].

## 4. Dielectric Tissue Modeling

Throughout the last decades, the four-pole Cole–Cole model is frequently used to model the dielectric behavior of biological tissue over a broad frequency range [19,42]:

(1) $$\epsilon =\epsilon_{\infty }+\sum_{n=1}^{4}\frac{\Delta \epsilon_{n}}{1+{\left(j2\pi f\tau_{n}\right)}^{\left(1-\alpha_{n}\right)}}+\frac{\sigma_{s}}{j2\pi f\epsilon_{0}}.$$

Furthermore, the Cole–Cole parameters can depend on tissue temperature, as first proposed by M. Lazebnik et al. for liver tissue [21] and later extended to blood, muscle, and fat [24,43]. This work conducted measurements in the 0.5–40 GHz frequency range. Here, the γ dispersion is dominant [44] and clearly visible in the presented data (Figure 5). Furthermore, the tail of the δ dispersion is present and causes the steep decrease in permittivity for increasing frequency that can be observed in the 0.5–2 GHz frequency range. Therefore, it sounds reasonable to fit the measured data with a two-pole Cole–Cole model. Nevertheless, a single-pole Cole–Cole model will be presented since the measured frequency range does not offer the required information to properly model the second dispersion. The δ dispersion is present in the 0.1 MHz to 2 GHz range (${\left(2\pi \tau_{2}\right)}^{-1}=0.45\;\mathrm{MHz}$) compared to the measurement range of the 0.5–40 GHz, and it causes a change from 7000 units of permittivity ($\Delta \epsilon_{2}=7000$) compared to the monitored difference of only 10 units in the tail [19]. Therefore, the obtained information regarding the δ dispersion is, in our opinion, too scarce to accurately represent it. It would be possible to fit this dispersion, but, in our opinion, it would be a purely numerical operation. Moreover, the fitting would be numerically unstable and would produce fitting parameters ($\Delta \epsilon_{2}$, $\tau_{2}$, and $\alpha_{2}$) with little to no physical meaning. As a consequence of ignoring this dispersion, the single-pole model will have a somewhat skewed γ dispersion to compensate for the missing δ dispersion. This translates in lower $\epsilon_{\infty }$, and higher $\Delta \epsilon_{1}$ and $\alpha_{1}$ values to compensate $\epsilon^{\prime }$. Similarly, an increase in $\sigma_{s}$ balances out the missing δ dispersion in $\epsilon^{\prime \prime }$.

**Figure 6:**
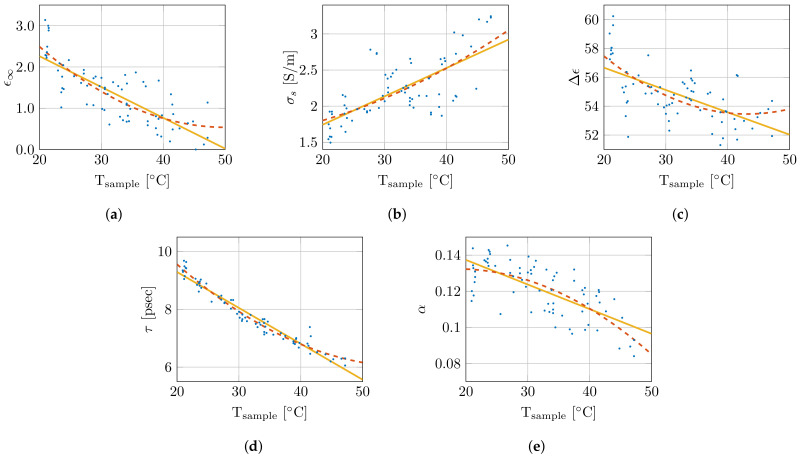
Overview of change in Cole–Cole parameters in function of sample temperature with a first (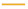) and second (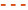) order polynomial fit. The Cole–Cole parameters $\epsilon_{\infty }$, $\sigma_{s}$, Δϵ, τ, and α are shown in (**a**), (**b**), (**c**), (**d**) and (**e**), respectively.

Therefore, the single-pole Cole–Cole model was selected and will be used throughout the remainder of this work. The nonlinear least-squares optimizer from MATLAB, using the Levenberg–Marquardt algorithm, simultaneously fitted the real and imaginary permittivity data for each measurement while optimizing the five single-pole Cole–Cole parameters ($\epsilon_{\infty }$, $\sigma_{s}$, Δϵ, τ, and α) [45]. The extracted Cole–Cole parameters are presented as a function of sample temperature in Figure 6. Additionally, they are fitted with a first ($p_{1}\times T+p_{0}$) and second order polynomial ($q_{2}\times T^{2}+q_{1}\times T+q_{0}$), which are added in the same figure. The corresponding polynomial parameters are given in Table 1. As is clear from Figure 6, the small coefficients $q_{2}$ of the quadratic term, and the limited difference in the $R^{2}$ values, the added complexity of the second order polynomial is not worth the limited improvement in the obtained fit. Therefore, a linear fit as function of sample temperature for the Cole–Cole parameters will be used from here on. This results in the following temperature-dependent dielectric model:

(2) $$\epsilon (f,T)=\epsilon^{\prime }\left(f,T\right)-j\epsilon^{\prime \prime }\left(f,T\right)$$

(3) $$\begin{array}{cc} \; & =\epsilon_{\infty }\left(T\right)\;+\frac{\Delta \epsilon \left(T\right)}{1\;+\;{\left(j2\pi f\tau \left(T\right)\right)}^{\left(1-\alpha \left(T\right)\right)}}+\frac{\sigma_{s}\left(T\right)}{j2\pi f\epsilon_{0}}\\ \; & =\;\left[p_{1,\epsilon_{\infty }}\times T+p_{0,\epsilon_{\infty }}\right] \end{array}$$

(4) $$\begin{array}{cc} \; & \;\;\;+\frac{\left[p_{1,\Delta \epsilon }\;\times \;T\;+\;p_{0,\Delta \epsilon }\right]}{1\;+\;{\left(j2\pi f\left[p_{1,\tau }\;\times \;T\;+\;p_{0,\tau }\right]\right)}^{\left(1-\left[p_{1,\alpha }\times T+p_{0,\alpha }\right]\right)}}\\ \; & \;\;\;+\frac{\left[p_{1,\sigma_{s}}\;\times \;T\;+\;p_{0,\sigma_{s}}\right]}{j2\pi f\epsilon_{0}}. \end{array}$$

**Figure 7:**
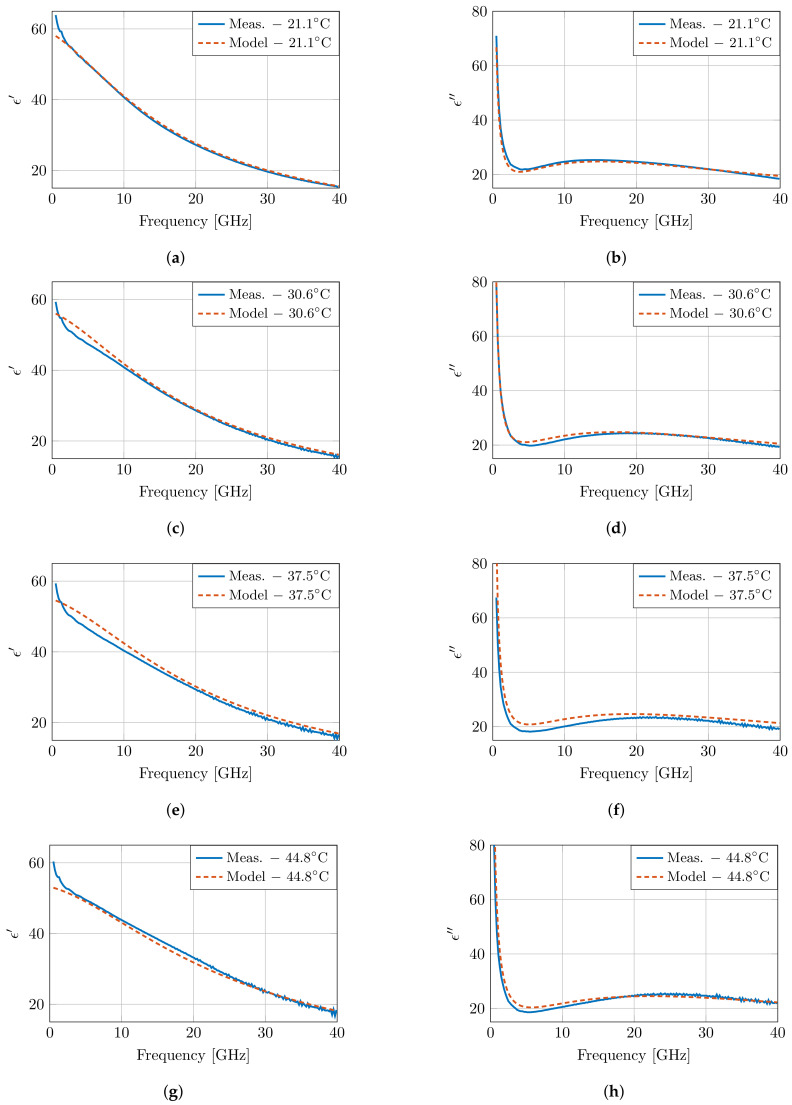
The dielectric properties of muscle tissue as proposed by the presented model and compared to the measurements at four temperatures as presented in Figure 5. The real and imaginary part are displayed in (**a**), (**c**), (**e**), (**g**) and (**b**), (**d**), (**f**), (**h**), respectively.

Figure 7 presents the modeled dielectric properties at four temperatures, and the results are very close to the measured properties in Figure 5. However, at low frequencies, a discrepancy is present between the model and the measurements. Due to the lack of a second dispersion in the presented model, the steep decrease in the measurements cannot be captured in the model. Therefore, a second order fit was performed following the same approach as specified above. Both models have a similar error overall, apart from the clear difference at low frequencies in the first order model due to the absence of the δ dispersion. This improvement is also present when evaluating the root mean square error (RMSE) of the real and imaginary part of the permittivity over all frequencies for all measurements. An RMSE of 0.7354 is obtained for the first order model, whereas this RMSE is reduced to 0.3144 in the second order model. However, this improvement comes at the cost of increased numerical instability, and the presented parameters would have little to no physical meaning.

In addition, the aforementioned crossover frequency of the model is clearly present around 6.5 GHz. However, due to the conciseness of the model, the crossover appears at a single frequency point, whereas a broader crossover zone is observed in measurements in the 6.5–10 GHz frequency band. Nevertheless, the temperature-dependent γ dispersion of pure water exhibits a similar broad crossover zone [7]; therefore, this broader crossover zone is expected, as well for high-water content tissues, such as muscle tissue, in this work.

**Figure 8:**
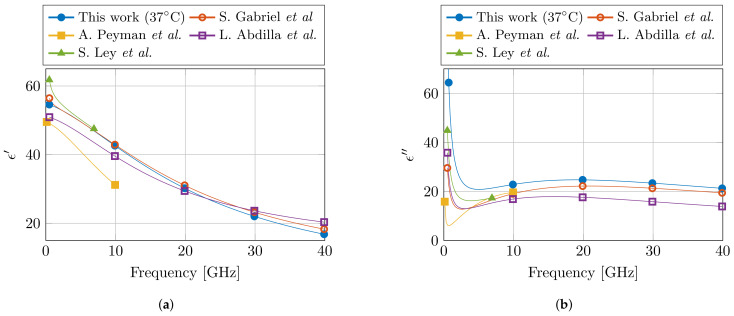
Comparison of the proposed dielectric model of muscle tissue at 37 °C with the models from literature in the corresponding frequency range. The real and imaginary part of the permittivity are shown in (**a**,**b**), respectively. (Literature data from S. Gabriel et al. [19], A. Peyman et al. [22], L. Abdilla et al. [23], and S. Ley et al. [24].)

Lastly, the obtained Cole–Cole parameters are compared with the available literature for dielectric properties of muscle at 37 °C. In the relevant frequency range, three Cole–Cole models were found at 37 °C [19,22,23], whereas a fourth model presented a temperature-dependent model in the 30–50 °C temperature range [24]. The relevant Cole–Cole parameters are presented in Table 2, and the models are compared in Figure 8. For the real part of the permittivity $\epsilon^{\prime }$, a good agreement is observed between this model and the models in literature. However, due to the incorporation of the δ dispersion in the models of [19,24], these two models do feature the steep decrease in $\epsilon^{\prime }$ that can be observed in the lower part of the frequency spectrum. In the case of the imaginary part of the permittivity $\epsilon^{\prime \prime }$, the presented model deviates from literature, and a higher value is observed.

## 5. Discussion on Measurement Improvements with Controlled Measurement Setup

The presented controlled measurement setup is, to the authors’ best knowledge, the first to actively control and monitor several confounders, e.g., sample temperature, sample dehydration, and probe-to-tissue contact pressure, and environmental conditions, e.g., room humidity and room temperature. Therefore, it is of interest to the computational life sciences community to discuss and evaluate whether the additional efforts result in more accurate dielectric measurements of biological tissue. With the implemented dehydration countermeasures, we noticed little to no tissue dehydration for temperatures below 40 °C, whereas, under normal lab conditions, dehydration would be present in this temperature range [30]. Due to the absence of tissue dehydration, the dielectric properties are better preserved, which is a first improvement of the presented controlled measurement setup. Secondly, a limited standard deviation of 0.61 kPa over all measurements was obtained with the PI-controlled lifting platform. This deviation in contact pressure could cause a change of −0.19% in the complex dielectric properties of the sample [31]. This change is comparable to or smaller than the complex uncertainty $s_{\mathrm{comb}.}$ of the measurement system (Figure 4). Therefore, the controlled measurement setup demonstrated that the obtained differences in dielectric measurements are independent of the applied contact pressure during measurements. This is a second clear improvement of this setup compared to the more commonly used manual lifting platform without any contact pressure feedback. Furthermore, the setup allows the measurement of other environmental conditions, which have been proven stable throughout all measurements.

However, in literature, several different reported dielectric models for muscle tissue can be found. This makes it challenging to objectively claim which model represents the dielectric properties of muscle tissue the best. Nevertheless, it is known that the dielectric model of S. Gabriel et al. [19] is a combination of a broad literature survey [17] and a measurement campaign [18]. Therefore, one could suspect that in this model the influence of aforementioned confounders over all different measurement setups and over all different human operators should be averaged out. Thus, this model [19] should approach the dielectric properties of muscle tissue the closest. Moreover, it is also the one that represents the dielectric properties of muscle tissue in the online database by the IT’IS Foundation [20]. Given the comparison at 37 °C between different models in Figure 8, we can observe the close agreement between our proposed model and the one from S. Gabriel et al. [19]. We believe that this close agreement is directly related to the carefully implemented precautions in the presented controlled measurement setup. However, more measurement campaigns on different tissues are required to confirm this statement. Nevertheless, in the meantime, the authors strongly suggest to incorporate the discussed precautions in other measurement campaigns in the community, and to report them along with the published data and models. This will only help to improve the stability of dielectric measurements of biological tissue, as well as aid in the evaluation of dielectric models of a single tissue type during comparison studies.

## 6. Conclusions and Future Work

This work presented two different research objectives. First, building upon the experience of our previous work, a controlled measurement setup for dielectric measurements of biological tissues is presented. Throughout the performed measurements, stable environmental conditions and a constant probe-to-tissue contact pressure were recorded. Furthermore, the dehydration countermeasures resulted in close to no relative weight loss for samples at a temperature below 40 °C and up to a maximum weight loss of 6% for samples at temperatures above 40 °C. With the carefully implemented precautions, a close agreement between our measurements and the one in literature has been obtained.

Secondly, this work conducted measurements on porcine muscle tissue in the 0.5–40 GHz frequency range with temperatures ranging from 20 °C to 45 °C. A single-pole Cole–Cole model was introduced to fit these measurements at each temperature. Afterwards, a first order polynomial was presented for each of the five Cole–Cole parameters ($\epsilon_{\infty }$, $\sigma_{s}$, Δϵ, τ, and α) as a function of sample temperature. With these temperature-dependent Cole–Cole parameters, a temperature-dependent dielectric model was proposed for 0.5–40 GHz frequencies and temperatures ranging from 20 °C to 45 °C. The model was compared with models available in the literature at 37 °C, and a good agreement was observed. With this model, dielectric data for muscle tissue can be generated over a broad frequency range for the design of in vivo diagnostic and therapeutic applicators. Nevertheless, future work should pursue the measurement of multiple tissue types following the MINDER framework and implementing the discussed precautions to limit the influence of external confounders. In addition, apart from different healthy tissues, ultra-wideband temperature-dependent dielectric properties of cancerous tissues should be gathered. Their dielectric profile will prove to be invaluable in the design of applicators for cancer hyperthermia and ablation procedures.

## Figures and Tables

**Table 1:** Parameters of the polynomial fit of the Cole–Cole parameters for the first ($p_{1}\times T+p_{0}$) and second order ($q_{2}\times T^{2}+q_{1}\times T+q_{0}$) with the corresponding goodness of fit values ($R^{2}$).

	$p_{1}$	$p_{0}$	$R_{\mathrm{first}}^{2}$	$q_{2}$	$q_{1}$	$q_{0}$	$R_{\mathrm{second}}^{2}$
$\epsilon_{\infty }$	−0.0746	3.7529	0.6257	0.0022	−0.2172	5.9668	0.6550
$\sigma_{s}$ [S/m]	0.0393	0.9585	0.5001	0.0005	0.0033	1.5166	0.5055
Δϵ	−0.1545	59.7534	0.3852	0.0075	−0.6453	67.3747	0.4351
τ [psec]	−0.1243	11.7765	0.9432	0.0025	−0.2883	14.3239	0.9643
α	−0.0014	0.1646	0.4924	−4.77·10${}^{-5}$	0.0018	0.1159	0.5259

**Table 2:** Overview of the Cole–Cole parameters for muscle tissue at 37 °C compared with the ones from literature. (Notice the absence of the second relaxation parameters for the single-pole Cole–Cole models in line 2, 3, and 5.)

Reference	$\epsilon_{\infty }$	$\sigma_{s}$ [S/m]	$\Delta \epsilon_{1}$	$\tau_{1}$ [psec]	$\alpha_{1}$	$\Delta \epsilon_{2}$	$\tau_{2}$ [nsec]	$\alpha_{2}$
S. Gabriel et al. [19]	4.00	0.20	50.00	7.23	0.10	7000	353.68	0.10
A. Peyman et al. [22]	3.00	0.11	46.64	12.21	0.10	-	-	-
L. Abdilla et al. [23]	11.78	0.95	39.45	9.17	0.10	-	-	-
S. Ley et al. [24]	4.75	0.53	50.73	6.62	0.18	7000	311.03	0.18
This work (37 °C)	1.23	2.41	53.73	7.21	0.11	-	-	-

## Data Availability

Not applicable.

## Abbreviations

The following abbreviations are used in this manuscript:

DIW	distilled water
EM	electromagnetic
IFBW	intermediate frequency bandwidth
ISM	industrial, scientific, and medical
IT’IS	foundation for research on information technologies in society
MINDER	minimum information for dielectric measurements of biological tissues [33]
PI	proportional-integral
PMMA	polymethylmethacrylate
RF	radiofrequency
RMSE	root mean square error
SHF	super-high frequency
VNA	vector network analyzer
UHF	ultra-high frequency
